# Remarks on the Early Symptoms and Treatment of Pott’s Disease of the Spine

**Published:** 1870-10

**Authors:** J. A. Wood

**Affiliations:** New York


### Remarks on the Early Symptoms and Treatment of Pott's Disease of the Spine.

BY J. A. WOOD, M. D., NEW YORK.

Some years since (March, 1858,) I reported in the New York *Journal of Medicine*, several cases of Pott's disease of the spine, treated with mechanical appliances and the internal use of medical agents, to the entire exclusion of setons, issues, and every other form of counter-irritation, and regardless even of the recumbent position.

In that journal I also gave a description, with illustrations, of the apparatus used in the treatment of the cases, a majority of which recovered, with the curvature completely reduced, although quite prominent when the treatment commenced.

Some of the patients had been confined to the bed several months, totally unable to sit up, wearied and wasted from the effects of the disease. One of them, with a bold angular curvature of the spine, having for its centre the last dorsal vertebra, with severe bed-sores over both trochanters, had become very much emaciated, and could not be turned in bed without convulsions. So great was the irregularity of the surface from extreme emaciation that it became necessary to envelop the body in cotton before the apparatus, or corset, could be advantageously applied. No spasm ever occurred after its first adjustment, the bed-sores soon healed, the health, strength, and flesh, were gradually restored, and the curvature entirely reduced.

The balance of the cases then reported were of longer standing, where a large amount of bony substance had been removed by disease, and consolidation to a considerable extent had taken place. Still, they were much improved in health, strength, and general figure.

Since that report, over four hundred and seventy additional cases have been treated in like manner, and generally with similar results. About eighteen per cent. of the whole number were affected with paralysis of the lower extremeties, and nearly twenty per cent. with abscess, both occurring at an earlier or later stage of the disease, which is met with at all periods of life from early infancy to old age.

The oldest person, however, that has come under my observation, affected with this disease, was aged fifty-five years. Another, in reference to whose case I was consulted by letter, was sixty years of age, and formerly president of one of our Western colleges, and was discharging the duties of that office when attacked with the disease. A more recent case, in a person fifty years of age, is now under treatment.

It occurs more frequently, however, in children under ten years old; but from two to five years of age may be considered the period of its most frequent occurrence.

The disease was often preceded by scarlet fever, whooping cough or measles; and children who had suffered from a severe attack of the former are very liable to fall into a state of permanently impaired health, and become a prey to some of the various chronic forms of scrofula, among the more serious of which is caries of the vertebræ.

Measles, also, in children and young persons of a scrofulous diathesis, frequently awaken the slumbering germs of that fearful malady, Pott's disease; while whooping-cough acts only mechanically upon the system in developing more rapidly the disease already existing, but not detected, perhaps, by any of its characteristic symptoms when the cough commenced. In a few instances the disease succeeded severe and protracted typhoid fever; first manifesting itself when the system, from its reduced condition, was comparatively disarmed of all power of resistance to the development of any hereditary or constitutional taint that might exist, as is frequently the case with incipient phthisis.

From the commencement of the disease up to that period when the curvature first made its appearance (usually in the form of a small knuckle), the average length of time did not vary much from ten months, and was often characterized by paroxysms of most acute suffering. When paralysis of the lower extremeties occurred, the recovery of the patient from his paralytic condition, under the treatment, was only a question of time, and that, often, of brief duration. When long protracted, it was more generally he result of imprudence and sometimes obstinacy of the patient, in persisting in too much exercise upon the feet when first commencing to walk.

In one instance, the patient, of a restive habit, had nearly recovered from the second attack when he fell from a considerable height and became the third time paralyzed, from which he has not regained, and probably never will regain, the use of his limbs.

Paralysis did not, I think, exist in one instance where the disease was situated below the last dorsal vertebra; but it occurred in an increased ratio, proceeding upward from that point. Neither was there a single case of it in the upper extremities connected with *genuine* Pott's disease. Such cases are very rarely found on record. It did occur, however, in one or both arms in the case of the patient sixty years old already alluded to, as having caries in the cervical region, throwing the head forward and downward with the chin resting on the sternum. This deformity was attended with severe and incessant pain, over which opiates, as I was informed by the attending physician, although liberally administered, appeared to have little or no control.

The treatment in those cases, in addition to the mechanical support, consisted of dry friction applied to the back and limbs, with flannel, or the bare hand, and the use, sometimes, of the galvanic battery. The loss of the power of locomotion, as a contingent of this disease, may be viewed as comparatively of minor importance. The patient is very sure to regain the use of his limbs under treatment. With a restive disposition the paralysis sometimes proves an advantage, as too much exercise upon the feet interferes with the efforts to reduce the curvature, and renders the ultimate success of the treatment less certain.

Dr. Pott ascribed his success in the treatment of paralysis of the lower extremities in this disease to the use of issues applied near the affected portion of the spine, and recommended their continuance for several months after the patient had recovered from his paralytic condition. It may be well to consider whether the remedy here recommended possesses merit superior to every other in such cases. Of this there appears to be no direct proof; and if the fact cannot be clearly substantiated by practical results, such practice should be discarded and treated as a source of unnecessary pain and suffering to the patient.

Abscesses sometimes created but little constitutional disturbance; neither did they in many instances appear to affect materially the ultimate results of the treatment, as ten or twelve only of the whole number thus affected terminated fatally, and those were generally of a most decidedly strumous character. In some instances the abscess terminated by absorption. This was more frequently the case when their locality was such as to subject them to the pressure of the corset. That result is very desirable whenever possible to effect it in any way, as it saves the patient from much discomfort, and, at least, *temporary* physical prostration and the attendants from an unpleasant and protracted duty, as the discharge seldom ceases until consolidation of the affected portion of the spine is far advanced. A premature use of the lancet, when abscess is the result of caries of the spine, is more frequently attended with serious constitutional results than when its contents are permitted to escape by a spontaneous opening.

If an abscess is quite painful it may be better, perhaps, to give early exit to the pus, even at a greater risk of constitutional irritation; and, if the surrounding tissues are likely to become too deeply involved by the further expansion of its walls, as is sometimes the case, the use of the lancet is imperative. Otherwise, it is better, usually, that the abscess should remain unmolested until its contents have approached near to the surface.

Very many of the cases presented for special treatment were of long standing, with marked deformity, impaired health, and general prostration, some having been subjected to one form of treatment and some to another. The seton, moxa, and various other forms of counter-irritation, had been resorted to, while in many cases the recumbent position was strictly enforced, in some instances, for a period of nine, twelve, and fifteen months, the patient not being permitted to rise from this position, even when taking nourishment.

But these different methods of treatment have all failed to accomplish what has often been effected, unattended with pain and suffering to the patient, by appropriate mechanical appliances in connection with a liberally sustaining diet and the use of such medicinal agents as the case appeared to demand, while the patient was comparatively unrestrained from air and exercise during the treatment.

Among the earlier symptoms of the disease is the manifest necessity for support, indicated by the patient's constant and instinctive inclination to seek it in leaning or throwing himself upon whatever may chance to come within his reach that will afford such support; and the more perfect is the design and adaptation of the support and the more promptly and skilfully it is adjusted and readjusted, the better for the patient, and the more successful and satisfactory will be the ultimate results of the treatment.—*N. Y. Med. Journal.*

